# Prevalence and risk factors of oral potentially malignant disorders in Indonesia: a cross-sectional study undertaken in 5 provinces

**DOI:** 10.1038/s41598-024-54410-4

**Published:** 2024-03-04

**Authors:** Elizabeth Fitriana Sari, Newell W. Johnson, Michael John McCullough, Nicola Cirillo

**Affiliations:** 1 Dentistry and Oral Health Discipline, Department of Rural Clinical Science, La Trobe Rural Health School, Bendigo, 3552 Australia; 2 Melbourne Dental School, The University of Melbourne, 720, Swanston Street, Carlton, VIC 3053 Australia; 3 Faculty of Dentistry, Universitas Padjadjaran, 45363 Bandung, Indonesia; 4 Menzies Health Institute QueenslandSchool of Medicine and Dentistry, Griffith University, Gold Coast, QLD Australia; 5 Faculty of Dentistry Oral and Craniofacial Sciences, King’s College London, London, UK

**Keywords:** OPMD, Oral cancer, Prevalence early detection, Risk factors, Cancer, Diseases, Oral cancer

## Abstract

Detection of subjects with oral potentially malignant disorders in a population is key to early detection of oral cancer (OC) with consequent reduction of cancer-related morbidity and mortality. Our aim was to investigate the prevalence and associated risk factors for OPMD in representative provinces of Indonesia. This cross-sectional study was undertaken in five Indonesian provinces: West Java (WJ), Jakarta (JKT), West Papua (WP), West Kalimantan (WK) and Banda Aceh (BA). Respondents answered a previously validated questionnaire including information on ethnicity, occupation, socioeconomic status (SES), oral health practices, and behaviours associated with oral cancer. An oral examination was undertaken using WHO standardized methodology. Data were analysed using ANOVA, Chi-Square, and logistic regression to assess association between risk factors and mucosal disease. A total of 973 respondents between the ages of 17 and 82 years was enrolled (WJ 35.5%,JKT 13.3% WP 18.3%, WK 9%, BA 23.9%). Tobacco smoking (14.8%), Betel quid (BQ) chewing (12.6%) and alcohol drinking (4%) varied geographically. A well-established OPMD was detected in 137 (14.1%) respondents and 2 (0.2%) presented with chronic ulceration later diagnosed as OC. Leukoplakia was the most common OPMD found (9.7%), while the prevalence of oral submucous fibrosis (OSMF), not previously described in the nation, was 2.3%. Poor knowledge of OC risk factors, poor oral hygiene behaviours, low-income SES and ethnicity were significantly associated with the presence of an OPMD. There is a previously under-reported high prevalence of OPMD in Indonesia. Overall, we found a strong correlation between the presence of an OPMD and individual habituation to known risk factors.

## Introduction

The increase in the incidence of Head and Neck Squamous Cell Carcinomata (HNSCC)—the 6th most common group of malignant neoplasms worldwide—has long been a serious global public health challenge^1^. More than 90% of HNSCCs involve the mucosal surfaces of the oral cavity, oropharynx and larynx and account for 3% of all cancers. In some developing countries such as India, Pakistan, Sri Lanka, and Taiwan, oral squamous cell carcinoma (OSCC) is the most common type of malignancy^2,3^. Whilst incidence numbers reported by WHO are the best available, these figures are estimates only and should be used with caution. Data collection in some countries is problematic: for example, epidemiological studies on oral cancer (OC) in geographically diverse areas have not been undertaken in Indonesia. This does not allow an accurate picture of the prevalence or incidence of OC and oral potentially malignant disorders (OPMD) in Indonesia to be drawn. Additionally, only a few South East Asian^4^ countries, including Singapore, Thailand, Philippines, Vietnam and Malaysia have population-based cancer registries, while Indonesia has not yet developed this public health information system^5^.

Several risk factors for OPMD and OC have long been recognized: notably tobacco–both smoked and smokeless—heavy alcohol consumption, and areca nut consumed in numerous forms including in betel quid (BQ)^1^. In Western countries, the major risk factors are cigarette smoking and heavy alcohol drinking, whereas in Asian countries, the prominent risk factors are smokeless tobacco, smoking and BQ chewing^2,6^. In Indonesia, the use of smokeless tobacco and areca nut in various forms is very popular in specific areas, which will inevitably lead to an as yet unquantified prevalence of OPMD. In particular, BQ chewing is known to induce oral submucous fibrosis (OSMF), a serious and debilitating disorder which is associated with an increased risk of OC development^7–9^.

According to data from *Badan Pusat Statistik Indonesia* (Indonesian Department of Statistic Centre), the Indonesian population was 280,159,787 in 2022^10^.It has been indicated in Indonesian basic health research undertaken both in 2010 and 2013 that one-third of Indonesians are tobacco smokers (about 87.3 million people)^11–13^. This makes Indonesia a nation with one of the highest smoking rates in the world. It is also one of the biggest tobacco producers^11–13^. The areca nut chewing habit is known as “Menginang” in Bahasa Indonesia, which comes from the Javanese words *tempat kinang* (meaning the storage of “betel” quid): it is common throughout the world to use the misnomer “betel” for the endosperm of the fruit of the areca palm tree. Areca nut is commonly consumed across the globe wrapped in a leaf of the betel (*Piper betle L*.) vine–hence “betel quid”. Areca nut chewing habits are a “heritage culture” passed down through many generations^5,14^. These lifestyle practices bring Indonesians at high risk of developing OPMD and OC. Unfortunately, no accurate data exist on the prevalence of OPMD in Indonesia. Rates of oral cancer are also uncertain given the lack of a comprehensive national cancer registration system.

A clinically visible OPMD may precede malignant transformation at the same site or the development of a cancer elsewhere in the mouth^15,16^. It is known that early detection of OC is important because survival rates markedly improve when the oral lesion is treated at an early stage^17^. Detection of any OPMD is, therefore, key and this can be effectively achieved by population screening. Furthermore, given the accessibility of the oral cavity, “conventional oral examination (COE)” using good illumination with white light, and digital palpation of suspicious areas, is the most common method used^18–20^. It would be critically important to undertake oral screening in Indonesia, not only to enable early management but also to detect the prevalence and quantify risk factors for OPMDs and OC among our varied populations. Hence, this present study focused on detection of overt malignant and potentially malignant abnormalities in the oral cavity. It was conducted in selected diverse areas in Indonesia utilizing visual and physical conventional oral examination (COE). The results provide valuable information on the prevalence of OC and OPMD, of their risk factors, and of awareness amongst the public in Indonesia.

## Material and methods

### Study design and population

This cross-sectional study used questionnaire surveys and a physical assessment and was reported in compliance to the STROBE checklist. We adopted a multistage, stratified, cluster-sampling procedure, which considered geographical region, degree of urbanisation, economic development status, sex and age distributions^21^, as well as tobacco use, areca nut and BQ chewing habits as utilised by the Indonesian Basic Health Research surveys undertaken in 2010 and 2013^11–13^.

### Setting

Drawing from the results of these 2010 and 2013 surveys, we randomly selected five of the ten regions outlined in these reports^21^. These were: Banda Aceh, Bandung (West Java), Jakarta (special city district of Indonesia), Pontianak (West Kalimantan), and Sorong (West Papua) (Supplementary Fig. 1). The data were collected from September 2016 to January 2017.

### Participants

Patients attending community services or a hospital for dental treatment were invited to join the survey. Local dental associations facilitated the recruitment of the general public through local authorities. On the survey day, the participants were given a clear explanation about the research, the time required to fill in the questionnaire and to be examined, the benefits and risks of becoming participant. Participants received a copy of plain language statement form (Supplementary Doc 1). Those who freely consented to participate in this study were enrolled after signing their informed consent (Supplementary Doc 2).

### Study size

The target sample size was established using the formula *n* = Z^2^P(1–P)/d^2^, where *n* is the sample size, Z is the statistic corresponding to level of confidence, P is expected prevalence and d is precision (corresponding to effect size). The following assumptions were made: confidence interval (CI) was set at 95% (type I error rate of 0.05), and effect size at 5%. Prevalence of OPMD was assumed between 5 and 10% of the population, resulting in a minimum sample size of 73 to 139.

## Variables and data source

### Questionnaire for respondents

A questionnaire consisting of 23 questions was developed from existing literature and modified to suit the local situation^22–24^. This questionnaire underwent a validation process by a panel of senior researchers based in Indonesia to critically evaluate suitability, clarity, simplicity, understanding and sequencing of the questions. Appropriate modifications to the questionnaire were made before being utilized in the main survey (Supplementary Doc 3).

We conducted a pilot trial with a group of 10 patients in Bandung, West Java, Indonesia using test and re-test method. To complete the questionnaire converted to *Bahasa Indonesia* took 15 min and 3–5 min were needed to undertake COE. Therefore, a total of 20 min for each respondent was required.

### Conventional oral examination

The COE used a step-by step guideline from the British Columbia OC prevention program (BCCA) in 2008^25^. adopting nomenclature, definitions and classification of potentially malignant disorders of the oral mucosa extant at the time^26^. It should be noted these have been recently updated^27^ but changes do not materially affect the data reported here. While not falling in any OPMD category, non-healing oral ulcerations were also recorded. The COE was conducted by a team of examiner dentists who undertook an inter-examiner calibration training for COE prior to performing COE.

Whilst the gold standard for the definitive diagnosis of OPMD is histological examination, conventional oral examination (COE) is considered to be the best approach in oral cancer screening^28^. A COE consists of 8 steps including extraoral visual examination and palpation of locoregional lymph nodes, intraoral visual assesment using white light and palpation of suspect areas^25^.

### Ethical approval

Ethical approval was obtained from Faculty of Medicine University of Padjadjaran, Health Research Ethic Committee, Indonesia (reference number 701/UN6.C1.3/KEPK/PN/2016), and from The University of Melbourne (Ethics reference ID: 1748812). Our research was performed in accordance with relevant guidelines and regulations. Informed consent was attained from all participants, and participants' names as well as other Health Insurance Portability and Accountability Act of 1996 (HIPAA) identifiers have been removed from all sections of the manuscript, including supplementary information.

### Formal approval and arrangements

The formal approval for conducting large-scale nation-wide research was granted by Indonesian Ministry of Home Affairs, Directorate General of Politic and National Affairs with reference number 440.02/4097/Polpum followed by approval from each provincial government, and relevant local city government. Formal arrangements for the issue of a certificate of approval to undertake this survey were made with Indonesian Dental Association branch Pontianak—West Kalimantan, Sorong—West Papua, Bandung—West Java, Central Jakarta—Jakarta Special District, and Faculty of Dentistry Universitas Syiahwala, Banda Aceh.

### Data analysis

Completed questionnaires were stored in SurveyMonkey. Data were exported into Microsoft Excel. De-identified clinical data were coded manually into Microsoft Excel to allow statistical analysis using Minitab Express^©^ version 1.5.1. 2017 and R commander. The difference between means was determined using multiway analysis of variance (ANOVA), Chi-Square and binary logistic regression were used to assess association between individual risk factors and mucosal diseases. A multiple logistic regression model was built to account for the effect of independent variables simultaneously. Differences were considered significant where *P* ≤ 0.05.

## Results

### General assessment of OPMD among Indonesians

Respondents enrolled in this study were 973 with a mean age of 42 (range 17–82). Females predominated (n = 748; 76.9%) and 49% (n = 479) of respondents were unemployed. Only a minority of respondents (30%) had some knowledge of OC (Supplementary Table S1). In our cohort, 14.5% of respondents were smokers, 12.6% were betel quid chewers and 3.9% were alcohol drinkers (Supplementary Table S2). Notably, 137 (14.1%) of the 973 had at least one well established OPMD and 2 respondents (0.2%) presented with chronic ulceration diagnosed after biopsy as OC (Supplementary Table S3). We chose to include these suspicious chronic ulcers in the OPMD group for ease of reporting^29^.

### Assessment of OPMD: types, sites, risk factors and geographical region

A total of 87 females and 52 males had an OPMD (Table 1). Leukoplakia was most common, accounting for 67.6% of all cases (94/139). The second common type was OSMF (15.8%), followed by leukoplakia combined with OSMF (7.9%). The least common was erythroplakia, found in only one person (0.7%), while erythro-leukoplakia had a higher prevalence (4.3%). Two persons (1.4%, 2/139) who presented with a past history of OC had a large ulcer on their tongue, which was later diagnosed as recurrence of OC (Table 1). OPMD mostly appeared on a single site of oral mucosa, whereas only 20 (14.4%) of 139 OPMD occurred on multiple sites. In 2 (1.4%, 2/139) respondents, most oral mucosal surfaces were involved. The most affected site was buccal mucosa (71.2%), 6.7% were found on lateral tongue and 4.3% on gingiva (Table 2). BQ chewing (n = 59) was the most frequent habit associated with OPMDs compared to other typical risk factors, particularly smoking tobacco, and alcohol drinking: alcohol drinking was, however, the habit bearing the strongest association (OR = 12.2) (Table 3).

**Table 1:** Type of OPMD or OC.

OPMD/OC	N	(%) of Study Population	(%) of OPMD/OC Case	F	M	Age (SD)
Erythroplakia	1	0.1	0.7	1	0	26.8 ± 0.0
Erythro-leukoplakia	6	0.6	4.3	5	1	49.8 ± 9.1
Leukoplakia	94	9.7	67.6	46	48	44.9 ± 15.3
Leukoplakia + OSMF	11	1.1	7.9	11	0	49.0 ± 13.0
OSMF	22	2.3	15.8	2	1	47.2 ± 14.0
Lichen Planus	3	0.3	2.2	20	2	46.8 ± 9.2
Ulcer (diagnosed as OC)	2	0.2	1.4	2	0	45.7 ± 14.6
Total OPMD or OC	139	14.3	100	87	52	44.3 ± 12.5
Normal	834	85.7	NA	661	173	42.5 ± 15.5

**Table 2:** Site of OPMD or OC.

Site of affected oral mucosal	Site	Type	Frequency (n)	Percentage (%)
Single site	Gingiva	L	6	4.3
	Hard Palate	L	1	0.7
	Buccal	E	3	2.2
		L	72	51.8
		E-L	1	0.7
		L-OSMF	4	2.9
		LP	3	2.2
		OSMF	16	11.5
	***Buccal***		***99***	***71.2***
	Labial	L	2	1.4
	Lateral tongue	L	6	4.3
		U	2	1.4
		OSMF	1	0.7
	***Lateral tongue***		***9***	***6.7***
	Full Mouth	OSMF	2	1.4
Multiple sites	Buccal + FOM	E	3	2.2
	Buccal + Dorsal tongue	L	1	0.7
	Buccal + Gingiva	L	2	1.4
	Buccal + Hard Palate	L	1	0.7
	Buccal + Lateral Tongue	L	5	3.6
	Gingiva + FOM	L	1	0.7
	Gingiva + Lateral Tongue	L	1	0.7
	Labial + Buccal + Ventral	L	1	0.7
	Lateral Tongue + FOM	L	1	0.7
	Buccal + Lateral Tongue + Ventral	OSMF	1	0.7
	Buccal + Soft Palate	OSMF	1	0.7
	Labial + Buccal	OSMF	1	0.7
	Labial + Buccal + FOM	OSMF	1	0.7
	***Multiple sites***		***20***	***14.4***
			**139**	**100**

L: Leukoplakia, E: erythroplakia, E-L: Erythro-leukoplakia, OSMF: OSMF, U: Ulcer, LP: Lichen planus, L-OSMF: Leukoplakia and OSMF: FOM : Floor of the mouth.

**Table 3:** Association between smoking, drinking and BQ chewing habit and OPMD or OC.

Risk factors	OPMD		OR (95%CI)	P-Value
	Yes	No		
BQ (Yes)	59	64	**8.9 (5.8, 13.5)**	** < 0.05**
BQ (No)	80	770		
Smoking (Yes)	53	88	**5.2 (3.5, 7.9)**	** < 0.05**
Smoking (No)	86	746		
Alcohol Drinking (Yes)	24	14	**12.2 (6.2, 24.3)**	** < 0.05**
Alcohol Drinking (No)	115	820		

Significantly different values are in bold.

In our cohort, 60 (43.17%) of 139 respondents who had an OPMD lived in WP and the remaining 79 with OPMD spread diversely amongst the other 4 regions (Supplementary Fig. 2). A comparison of the presence of OPMD’s across the five regions showed that people living in WP had 22.3, 17.8, and 5.4-fold higher risk of having OPMD compared to people living in JKT, BA, and WJ respectively, but only 1.7 times higher risk of OPMD compared to people living in WK. This analysis of respondents showed that the highest OPMD rate occurred in people from WP (Table 4).

**Table 4:** Region and OPMD or OC.

Level A	Level B	Odds Ratio	95% CI	P-Value
WP	BA	**19.8**	**(9.5, 41.2)**	** < 0.05**
WP	WJ	**5.4**	**(3.3, 8.7)**	** < 0.05**
WP	JKT	**22.3**	**(7.7, 64.6)**	** < 0.05**
WP	WK	**1.7**	**(0.9, 3)**	** < 0.05**
WP	ALL REGIONS	**4.9**	**(3.3,7.2)**	** < 0.05**

Odds ratio for level A relative to level B.

Significantly different values are in bold.

### BQ Chewing and risk of OPMD

Individuals consuming BQ in any form were at an increased risk of presenting with an OPMD (OR = 8.9, 95% CI 5.8–13.5). The duration and frequency of BQ consumption in OPMD cases showed that respondents who had consumed BQ for more than 15 years were 2.5-fold more likely to develop OPMD compared to those who were BQ consumers for less than 15 years (Table 5). Respondent who chewed BQ regularly had 2.5-fold increased risk of OPMD occurrence compared to occasional users (Table 6). BQ in fabricated package was associated with an increased risk of OPMD (3.6-fold) compared to BQ that was prepared ad hoc from fresh areca nut, leaf/stem inflorescence and other spices (Table 7).

**Table 5:** The association of duration of BQ consumption with OPMD or OC.

Duration of consumption	OR (95%CI)	P value	Frequency (%)	Mean age (SD)
> 15 years *vs *all BQ users	2.5 (1.2, 5.2)	< 0.05	67 (54.5)	48.2 (15)
< 15 years *vs *all BQ users	0.4 (0.2, 0.8)	< 0.05	56 (45.5)	48.7 (16)
***Total***			***123 (100)***

**Table 6:** The association of frequency of BQ consumption with OPMD or OC.

Frequency of consumption	OR (95%CI)	P-value	Frequency (%)	Mean age (SD)
Regular User *vs *all BQ users	2.5 (1.4, 4.5)	< 0.05	62 (50.4)	47.3 (18)
Occasional User *vs* all BQ users	0.2 (0.06, 0.4)	< 0.05	44 (35.8)	50.1 (15)
***Total***			***123 (100)***

**Table 7:** BQ package and OPMD or OC.

BQ Package	OR	P-Value	Frequency (%)	Mean of Age (SD)
Yes	3.6 (1.5, 8.5)	< 0.05	**89 (72.4)**	**50.5 (15)**
No			**34 (27.6)**	**42.8 (15)**
***Total***			***123 (100)***	

Significantly different values are in bold.

The various ingredients of BQ consumed in different regions of Indonesia differed significantly: respondents who consumed BQ containing betel stem inflorescence (BQ-SI) had a risk of OPMD almost 4 times higher (OR = 4.2) compared to respondents who consumed BQ containing betel leaf (BQ-L). Importantly, BQ-SI was only consumed in WP, while BQ-L was consumed in BA, WK and WJ. As expected, the addition of tobacco to BQ-L chewing increased the risk (OR = 10.2) compared to consuming BQ-L without tobacco. Importantly, no chewers of betel leaf alone had an OPMD^30,31^ (Table 8).

**Table 8:** The association of various BQ ingredients with OPMD or OC.

Level A	Level B	Odds Ratio	95% CI	P-Value
BQ + tobacco	BQ-L	*10.2	**(1.7, 62.3)**	** < 0.05**
BQ-SI	BQ-L	*4.2	**(1.5, 11.6)**	** < 0.05**
BQ-SI + To	BQ-L	*1.3*	*(0.1, 10.2)*	*NS*
Betel leaf	BQ-L	*0.2*	*(0.02, 2.4)*	*NS*
Other	BQ-L	*0.2*	*(0.019, 1.9)*	*NS*
Not chewing BQ	BQ-L	**0.2**	**(0.09, 0.6)**	** < 0.05**
BQ-SI	BQ-L + Tobacco	*0.4*	*(0.06, 2.8)*	*NS*
BQ-SI	BQ-L + Tobacco	*0.1*	*(0.01, 1.5*	*NS*
Betel leaf	BQ-L + Tobacco	**0.0**	**(0.002, 0.3)**	** < 0.05**
Other	BQ-L + Tobacco	**0.0**	**(0.001, 0.3)**	** < 0.05**
Not chewing BQ	BQ-L + Tobacco	**0.0**	**(0.004, 0.1)**	** < 0.05**
BQ-SI + Tobacco	BQ-SI	*0.3*	*(0.04, 2.1)*	*NS*
Betel leaf	BQ-SI	**0.1**	**(0.006, 0.5)**	** < 0.05**
Other	BQ-SI	**0.0**	**(0.005, 0.4)**	** < 0.05**
Not chewing BQ	BQ-SI	**0.1**	**(0.03, 0.1)**	** < 0.05**
Betel leaf	BQ-SI + Tobacco	*0.2*	*(0.01, 3.1)*	*NS*
Other	BQ-SI + Tobacco	*0.1*	*(0.009, 2.5)*	*NS*
Not chewing BQ	BQ-SI + Tobacco	**0.2**	**(0.03, 1.1)**	**NS**
Other	Betel Leaf	*0.9*	*(0.04, 17.5)*	*NS*
Not chewing BQ	Betel Leaf	*1.0*	*(0.1, 8.9)*	*NS*
Not chewing BQ	Other	*1.2*	*(0.1, 9.7)*	*NS*

Odds ratio for level A relative to level B, NS = Not significant.

BQ-L = areca nut, slaked lime, and Betel Leaf.

BQ-SI = areca nut, slaked lime, and Betel Stem Inflorescence (only consumed in WP).

Others = other ingredients apart from areca nut, slaked lime, betel leaf, betel stem inflorescence and tobacco.

Significantly different values are in bold.

Not significantly different values are in italic.

### Cigarette smoking and risk of OPMD

Smoking was a significant risk factor for OPMD (OR = 5.2, 95% CI 3.5–7.9). The duration of smoking habit (less than 5 years, 5–15 years and more than 15 years) was not significantly related with OPMD (Table 9). However, in respondents who consumed more than 10 cigarettes a day the risk of having OPMD was almost tripled (OR = 2.7) compared to those who consumed less than 10 cigarettes (Table 10).

**Table 9:** Duration of smoking and OPMD or OC.

Level A	Level B	Odds ratio	95% CI	P-values
5–15 years	< 5 years	*1.1*	*(0.41, 3.02)*	*NS*
> 15 years	< 5 years	*1.1*	*(0.42, 2.66)*	*NS*
Non-smokers	< 5 years	**0.2**	**(0.1, 0.47)**	** < 0.05**
> 15 years	5–15 years	*1*	*(0.42, 2.15)*	*NS*
Non-smokers	5–15 years	**0.2**	**(0.09, 0.38)**	** < 0.05**
Non-smokers	> 15 years	**0.2**	**(0.12, 0.34)**	** < 0.05**

Odds ratio for level A relative to level B.

**Table 10:** The number of cigarettes consumed daily and OPMD or OC.

Level A	Level B	Odds ratio	95% CI	P-value
> 10	< 10	2.7	(1.33, 5.49)	< 0.05
Non-smokers	< 10	0.3	(0.18, 0.52)	< 0.05
Non-Smokers	> 10	0.1	(0.06, 0.20)	< 0.05

Odds ratio for level A relative to level B.

The occurrence of OPMD was not associated with the different types of cigarettes consumed overall. The prevalence of OPMD between non-smokers and the various types of cigarettes smoked showed non-smokers respondents had a much lower risk of having OPMD compared to kretek (original Indonesian cigarette) and filtered cigarette smokers, respectively (Table 11).

**Table 11:** The type of cigarette and OPMD or OC.

Level A	Level B	Odds ratio	95% CI	P-value
Filter	Kretek	*1.2*	*(0.5, 2.9)*	*NS*
Non-smokers	Kretek	**0.3**	**(0.1, 0.6)**	** < 0.05**
Non-smokers	Filter	**0.2**	**(0.1, 0.3)**	** < 0.05**

Odds ratio for level A relative to level B.

Significantly different values are in bold.

Not significantly different values are in italic.

### Alcohol drinking and risk of OPMD

When considering crude rates, alcohol drinkers had 12.2-fold increased risk of having OPMD compared to the non-drinkers (95% CI 6.2–24.3). In relation to duration of drinking alcoholic beverages, we found no significant correlation between duration of consuming alcohol with the occurrence of OPMD in oral mucosa (Table 12). Furthermore, the amount of alcohol beverage consumed in a day was not significantly correlated to OPMD either (Table 13). In addition, respondents who drank arak putih, vodka, mixed brands, and beer had 25.2, 21.9, 15.5, and 7.6-times higher risk of having OPMD compared to non-drinker respondents, respectively (Table 14).

**Table 12:** Duration of drinking alcohol and OPMD or OC.

Level A	Level B	Odds ratio	95% CI	P-value
5–15 years	< 5 years	*1.2*	*(0.09, 15.84)*	*NS*
> 15 years	< 5 years	*0.5*	*(0.12, 2.38)*	*NS*
Not drink	< 5 years	**0.1**	**(0.02, 0.21)**	** < 0.05**
> 15 years	5–15 years	*0.4*	*(0.04, 5.03)*	*NS*
Non-drinkers	5–15 years	**0.1**	**(0.005, 0.49)**	** < 0.05**
Non-drinkers	> 15 years	**0.1**	**(0.05, 0.28)**	** < 0.05**

Odds ratio for level A relative to level B.

Significantly different values are in bold.

Not significantly different values are in italic.

**Table 13:** The amount of alcohols consumed per day and OPMD or OC.

Level A	Level B	Odds Ratio	95% CI	P-Value
2–5 bottles	1 bottle	*2.2*	*(0.38, 12.71)*	*NS*
Non-drinkers	1 bottle	**0.09**	**(0.04, 0.21)**	** < 0.05**
Non-drinkers	2–5 bottles	**0.04**	**(0.009, 0.21)**	** < 0.05**

Odds ratio for level A relative to level B.

Significantly different values are in bold.

Not significantly different values are in italic.

**Table 14:** The different brand of alcohol beverages and OPMD or OC.

Level A	Level B	Odds Ratio	95% CI	P-Value
Cap tikus	Arak Putih	*4.1*	*(0.17, 98.77)*	*NS*
Vodka	Arak Putih	*1.2*	*(0.07, 20.52)*	*NS*
Beer	Arak Putih	*3.3*	*(0.55, 19.82)*	*NS*
Mix	Arak Putih	*1.6*	*(0.09, 28.69)*	*NS*
Vodka	Cap tikus	*0.3*	*(0.007, 11.32)*	*NS*
Beer	Cap tikus	*0.8*	*(0.04, 15.14)*	*NS*
Mix	Cap tikus	*0.4*	*(0.0099, 15.89)*	*NS*
Not drink	Cap tikus	*6.2*	*(0.38, 100.46)*	*NS*
Beer	Vodka	*2.9*	*(0.22, 37.99)*	*NS*
Mix	Vodka	*1.4*	*(0.046, 42.75)*	*NS*
Mix	Beer	*0.5*	*(0.04, 6.52)*	*NS*
Vodka	*Non-drinkers	**21.6**	*(1.93, 247.09)*	< *0.05*
Arak Putih	*Non-drinkers	**25.2**	*(5.33, 119.18)*	< *0.05*
Beer	*Non-drinkers	**﻿7.6**	*(2.999, 19.36)*	< *0.05*
Mix	*Non-drinkers	**﻿15.5**	*(1.37, 176.24)*	< *0.05*

Odds ratio for level A relative to level B, NS = Not significant.

Significantly different values are in bold.

Not significantly different values are in italic.

### Knowledge of risk factors, oral health behaviours and OPMD

People who had awareness of OC risk factors were more likely not to have OPMD (OR = 0.18), as opposed to those who did not have any knowledge (Table 15). Only 17.78% were educated by a dentist, and as high as 82.22% admitted that dentists did not give any information about OC. However, respondents that had been educated by dentists had a significantly reduced risk (OR = 0.5, 95% CI 0.27, 0.85) of OPMD whereas those who learned about OC from other sources did not (Table 15).

**Table 15:** Knowledge of risk factors and OPMD or OC.

Knowledge of OC	OPMD		OR (95%CI)	P-value
	Yes	No		
Have knowledge (Yes)	7	191	0.2 (0.08, 0.39)	< 0.05
Have knowledge (no)	132	643		
Knowledge from dentist (yes)	14	159	0.5 (0.27, 0.85)	< 0.05
Knowledge from dentist no)	125	675		
Knowledge from government (yes)	15	105	*0.8 (0.47, 1.49)*	*﻿0.37*
Knowledge from government (No)	124	729		

Significantly different values are in bold.

Not significantly different values are in italic.

Those who responded that they brushed their teeth properly had a reduced risk of OPMD (OR = 0.6) compared to those who did not know how to brush their teeth (Table 16). Brushing teeth at least twice a day (in the morning and before going to bed), the teeth being brushed for at least 2 min, and all of the tooth surfaces were cleansed was considered as proper brushing for this study. In addition, respondents who stated that they brushed their teeth twice a day had lower rates of OPMD compared to those brushing their teeth once a day (Table 17).

**Table 16:** Oral Health Behaviour and OPMD or OC.

Oral health behaviour		OPMD or OC		OR (95%CI)	P-value
		Yes	No		
Brush teeth	Yes	132	779	*1.3 (0.6, 3.5)*	*0.2*
	No	7	55		
Proper toothbrush	Yes	71	524	**0.6 (0.43, 0.89)**	** < 0.05**
	No	68	310		

Significantly different values are in bold.

Not significantly different values are in italic.

**Table 17:** Frequency of tooth brushing per day and OPMD or OC.

Level A	Level B	Odds ratio	95% CI	P-value
Twice	Once	*0.6	**(0.33, 0.98)**	** < 0.05**
Thrice or more	Once	*0.7*	*(0.39, 1.38)*	*NS*
Thrice or more	Twice	*1.3*	*(0.84, 2.05)*	*NS*

Odds ratio for level A relative to level B, NS = Not significant.

Significantly different values are in bold.

Not significantly different values are in italic.

### Ethnicity, Socioeconomic status (SES), and OPMD

When the risk of development of OPMD in various ethnicities was assessed, the results showed that Papua (WP region) and Dayak (WK region) ethnicities had 23.6 and 21.3-fold higher risk compared to Aceh ethnicity (BA region). Javanese, Balinese and East Nusa Tenggaranese (ENT) did not have significant risk differences compared to Aceh ethnic groups (Table 18). Conversely, Sulawesi and Maluku ethnicities showed 7 times higher risk than Aceh ethnicity.

**Table 18:** Ethnicity and OPMD or OC.

Level A	Level B	Odds ratio	95% CI	P-Value
Dayak	Aceh	**21.3**	**(8.81, 51.64)**	** < 0.05**
Papua	Aceh	**23.6**	**(11.11, 50.2)**	** < 0.05**
North and West Sumatra	Aceh	*1.5*	*(0.51, 4.61)*	*NS*
Java, Bali, ENT	Aceh	**2.6**	**(1.32, 5.29)**	** < 0.05**
Sulawesi and Maluku	Aceh	**7.0**	**(2.76, 17.83)**	** < 0.05**
Papua	Dayak	*1.1*	*(0.51, 2.41)*	*NS*
North and West Sumatra	Dayak	**0.1**	**(0.024, 0.22)**	** < 0.05**
Java, Bali, ENT	Dayak	**0.1**	**(0.06, 0.25)**	** < 0.05**
Sulawesi and Maluku	Dayak	**0.3**	**(0.13, 0.85)**	** < 0.05**
North and West Sumatra	Papua	**0.1**	**(0.024, 0.18)**	** < 0.05**
Java, Bali, ENT	Papua	**0.1**	**(0.07, 0.19)**	** < 0.05**
Sulawesi and Maluku	Papua	**0.3**	**(0.14, 0.64)**	** < 0.05**
Java, Bali, ENT	North and West Sumatra	**1.7**	**(0.66, 4.5)**	** < 0.05**
Sulawesi and Maluku	North and West Sumatra	**4.6**	**(1.47, 14.16)**	** < 0.05**
Sulawesi and Maluku	Java, Bali, ENT	**2.7**	**(1.26, 5.58)**	** < 0.05**

Odds ratio for level A relative to level B, NS = Not significant.

Significantly different values are in bold.

Not significantly different values are in italic.

SES of individuals was assessed based on location at which the COE was conducted, on occupation, health insurance status, and on ease of travel to dental health facilities. Location was either in the community service (CS) or *Puskesmas* or one of the study villages (VI), in both cases whether located in a rural or urban area. Respondents who were screened at urban-CS places had a lower risk (OR = 0.43) of OPMD compared to those screened at rural-CS (Table 19). Similarly, respondents screened at urban-VI also had reduced risk of OPMD compared to those screened at rural-VI. Respondents who were screened at the rural-CS had a 2.6 times increased risk of OPMD compared to urban-VI. Thus, respondents who were examined at the rural area had higher odds of being diagnosed with OPMD compared to the respondents screened at urban areas, whether it was screened in CS or VI (Table 19).

**Table 19:** SES and OPMD or OC.

SES variables	Level A	Level B	Odds Ratio	95% CI	P-Value
Location of COE	Rural-VI	Rural-CS	*0.9*	*(0.53, 1.5)*	*NS*
	Urban-VI	Rural-CS	**0.2**	**(0.1, 0.33)**	** < 0.05**
	Urban-CS	Rural-CS	**0.4**	**(0.27, 0.68)**	** < 0.05**
	Urban-VI	Rural-VI	**0.2**	**(0.1, 0.41)**	** < 0.05**
	Urban-CS	Rural-VI	**0.5**	**(0.27, 0.85)**	** < 0.05**
	Urban-CS	Urban-VI	**2.3**	**(1.19, 4.38)**	** < 0.05**
Occupation	Non-government employee	Government employee	**3.3**	**(1.8, 5.9)**	** < 0.05**
	Un-employed	Government employee	*1.5*	*(0.81, 2.69)*	*NS*
	Un-employed	Non-government employee	**0.5**	**(0.3, 0.67)**	** < 0.05**
Health Insurance	Non-Government	Government	**0.3**	**(0.2, 0.55)**	** < 0.05**
	Yourself	Government	*0.8*	*(0.5, 1.164)*	*NS*
	Yourself	Non-Government	**2.3**	**(1.30, 4.09)**	** < 0.05**
Travel to dental health facilities	Easy	Not easy	*1.6*	*(0.99, 2.63)*	*NS*

Significantly different values are in bold.

Not significantly different values in italic.

The occupation of respondents was grouped into government employee, non-government employee and un-employed. The total number of un-employed respondents were 479 (Female = 422, Male = 57). The family expenses or income were unable to measure as their incomes were unstable with varied occupations as farmer, fisher, seller in traditional market or government employee. The non-government employee respondents had a 3.3-fold higher risk of OPMD compared to government employees. Low-income association with the occurrence of OPMD was evident (Table 19).

Health insurance was grouped into those with government insurance, non-government insurance and self-funded health care costs. Indonesian government insurance is given strictly only to those in considerable financial need, with many terms and conditions. Respondents who had non-government insurance may be considered economically able and these had less risk (OR = 0.3) of having an OPMD compared to those who had government insurance.

Traveling to the dental health facilities was assessed from subjective responses as to whether they felt that such travel was easy or not. This was unrelated to the occurrence of an OPMD (Table 19).

### The relation between all putative risk factors and presence of an OPMD in a multiple regression model

Multiple regression analysis was carried out to analyse the relation between all risk factors, considered as independent variables, and OPMD. Smoking, BQ chewing, and ethnicity were, overall, the most significant risk factors associated with OPMD. Other significant associations were type of health insurance, type of alcohol, location of examination, knowledge of OC, and occupation respectively, as shown in Table 20. Gender, alcohol drinking, and brushing teeth daily were found to be not significant.

**Table 20:** All risk factors and OPMD or OC.

Risk factors	Coefficient	SE coefficient	95% CI	P-value
Constant	*0.629*	*0.191*	(*0.25, 1.006*)	*3.3*
Gender	− *0.02*	*0.0273*	*(− 0.07, 0.04)*	*0.5*
Age	*0.001*	*0.000*	*(− 0.00003, 0.003)*	*0.06*
Occupation	− **0.029**	0.015	(− **0.06**, − **0.0007**)	**0.05**
Health insurance	− **0.030**	**0.008**	**(− 0.05, − 0.01**)	**0.0005**
Smoking	**0.184**	**0.034**	**(0.12, 0.3)**	** < 0.0001**
Betel quid chewing	**0.304**	**0.0338**	**(0.2, 0.4)**	** < 0.0001**
Alcohol drinking	* − 0.078*	*0.121*	*(− 0.3, 0.2)*	*0.5*
Knowledge of oral cancer	** − 0.068**	**0.028**	**(− 0.1, − 0.01)**	**0.02**
Proper brushing teeth	* − 0.004*	*0.042*	*(− 0.09, 0.08)*	*0.9*
Type of alcohol	** − 0.098**	**0.035**	**(− 0.2, − 0.03)**	**0.006**
Ethnicity	**0.029**	**0.007**	**(0.02, 0.04)**	** < 0.0001**
Location of examination	** − 0.025**	**0.009**	**(− 0.04, − 0.006)**	**0.01**

Significant different values are in bold.

Not significant different values are in italic.

## Discussion

This is the first representative study of OPMD in Indonesia. It reveals a substantial prevalence of 14.3% (139/973 persons). Well-known risk factors, knowledge of risk factors, oral health behaviour, ethnicity and SES were significantly associated with the prevalence of OPMD.

This overall prevalence of OPMD is higher than described in studies using a similar method of subject acquisition described recently from Guam﻿^32^ and Japan^70,32^. It has previously been shown that the prevalence of oral lesions, as well as the reported prevalence of smoking and drinking, as observed in the community dental health services, closely represents their prevalence in the general population^33^. Thus, opportunistic screening in a dental practice setting appears to be a realistic alternative to population screening^33^.

In the present study, a strong association was observed between some risk factors and OPMD. The most common risk factor was smoking cigarettes (14.5%), followed by areca nut/betel quid chewing (12.6%) and alcohol consumption (3.9%). A region where all these 3 habits are prevalent is WP, and one of the most significant results of our study was the record of a worryingly high prevalence of OPMD in this region: sixty (44.1%) out of the 139 respondents who had OPMD were living in WP and the other 55.9% of OPMD spread diversely amongst the other 4 regions. WP has a strong culture in BQ chewing passed down through many generations as well as the drinking of traditionally made alcohol beverages. Conversely, people who live in JKT with better social economic status and healthy nutrition had the lowest prevalence of OPMD, with a better understanding of OC risk factors. Previous studies have also reported that high SES relates to a relatively low risk of having OC^34,35^. Consistently, people living in WJ had a relatively low rate of OPMD. Across the nation individuals living in urbanised regions are at a lower risk of developing OPMD. This may also be confounded by the fact that most of the people living in JKT, WJ and BA are Muslims and are therefore less likely to consume alcoholic beverages^36^, however after controlling for confounders the regression analysis showed that alcohol drinking on its own was not associated with increased risk of OPMD in these populations.

The most common affected site of OPMD was buccal mucosa (71.22%). Previous studies showed that approximately 70% of leukoplakias are found on buccal mucosa, lip vermilion, and gingiva, and yet lesions on buccal mucosa rarely show dysplastic or malignant change compared to lesions seen on the floor of the mouth, lip vermilion and gingivae^37^. OPMD found in the floor of the mouth (FOM) together with other sites were found in 6 respondents (4%). The lateral tongue and floor of the mouth are known to be high risk sites of OPMD because OPMDs in these locations are more likely to develop into malignancy. In the present study, we found lateral tongue and floor of the mouth sites were the second and third most common sites for OPMD. Two respondents with a large single ulcer on lateral border of the tongue were diagnosed with oral squamous cell carcinoma (OSCC) and subsequently treated. Our results suggest that the risk of developing OC from OPMD is not negligible in the Indonesian population and requires urgent management.

### Betel quid chewing and OPMD

Many epidemiological, clinical and laboratory studies have reported a strong correlation between BQ chewing and OPMD or OC, specifically OSMF and leukoplakia development in the oral cavity^38–42^. In our study, consuming BQ for more than 15 years was shown to be associated with a 2.5-time higher risk of having OPMD compared to a timeframe of less than 15 years. One of the OPMD caused by BQ chewing is OSMF, a chronic disease known to aggravate with time and in relation to the duration of BQ use^42^. In remote regions, it is common to consume BQ from an early of age, and some reported that this habit commences in individuals as young as 10 years old^41^. Respondents in the present study who chew BQ regularly everyday were more likely to have OPMD compared to occasional users. Consistently, the development and severity of OSMF is known to correlate to BQ use in a dosage, frequency, and time dependent manner^41^.

The various ingredients of BQ are also suspected to differentially influence OPMD development. Most BQ are composed of areca nut, slaked lime and betel leaf or betel SI, and some additional spices, tobacco, *Ungaria gambir*, or other components as a mixture in BQ chewing. Taiwanese and Melanesian commonly chose betel SI (flower or pods) rather than betel leaf^43^. In the present study, only people living in WP consumed BQ-SI and all are of Melanesian ethnicity. This custom of BQ-SI chewing exists in WP and is shared with Papua New Guinea. The Papua region (West Papua and Papua, Indonesia) covers the western part of New Guinea island, whereas the eastern part (Papua New Guinea, PNG) is a separate sovereign country. The indigenous population of both Papua and PNG are Melanesian^44^. Interestingly, logistic regression analysis showed that BQ-SI chewers had 4.2 times higher risk of having OPMD compared to BQ-L chewers. We also showed that none of the betel leaf chewers had OPMD in the oral cavity. Both betel leaf and betel SI alone have been shown to contain polyphenolic as antimutagenic compounds^43,45,46^. However, safrole is a major phenol found predominantly in betel SI and may act as carcinogen^46,47^.

Our study also confirmed that the addition of tobacco to BQ-L chewing increases the risk (OR = 10.2) of OPMD compared to consuming BQ-L alone. Several studies have convincingly demonstrated that tobacco increases the frequency of onset of OSMF and accelerates malignant change^48,49^ One study in Pakistan found that adding tobacco into BQ increased the severity of clinical grading of OSMF in a statistically significant manner^41^.

BQ in commercial packaged form was associated with a 3.6-time higher risk of OPMD compared to non-package BQ prepared by the users themselves. This “home-made” BQ was variably composed of fresh areca nut, leaf/SI, and additional spices and possibly possesses more polyphenols and other antimutagenics compared to fabricated BQ.

Further research is required to clearly correlate the presence of OPMD and the type of ingredients and fabricated BQ packages used in Indonesia.

### Cigarette smoking, alcohol drinking, and OPMD

Cigarette smokers had 5.22-time increased risk of OPMD compared to the non-smoker’s respondents. Tobacco has been proven as one of major risk factors associated with OPMD and OC^35,39,50^. In SEA countries such as Indonesia, smoking and BQ chewing are the most common risk factors associated with OC^5^. Further logistic regression analysis showed that differences in the duration of smoking did not correlate with OPMD prevalence. However, the number of cigarettes consumed (more or less than 10 a day) was shown to have had a significant impact (OR = 2.7) on the presence of OPMD. Further logistic regression analysis showed that differences in the duration of smoking did not correlate with OPMD prevalence. However, the number of cigarettes consumed (more or less than 10 a day) was shown to have had a significant impact (OR = 2.7) on the presence of OPMD.

The occurrence of OPMD was not significantly associated with the different type of cigarettes consumed. However, when comparing non-smokers to individuals smoking different types of cigarettes, non-smokers respondents had 3.8 and 5.3-times lower risk of having OPMD compared to kretek and filter cigarette smokers, respectively.

There are two major types of cigarettes in Indonesia, which are kretek and filter. Kretek is characterised by the use of a significant amount of clove, the sun-dried flower buds of the clove tree (*Syzygium aromaticum, syn. Eugenia aromaticum*), added to the tobacco since the early 1880s. The clove content of the kretek cigarettes varies typically between 20 and 40% (w/w). Kretek are mostly made from domestic tobaccos especially from Java, Bali, Sumatra, and Lombok^51^. There are more than 20 tobacco varieties blended in a kretek. Kretek itself comes from the word that imitates the sound produced by object when it’s burned, the cracking noise made by clove when it’s burned:”kretek kretek^51,52^^’^. Conversely, filter cigarettes do not contain clove but only tobaccos with lower tar and nicotine level compared to kretek^51^. Six in vitro and in vivo studies reported that Indonesian kretek cigarettes unexpectedly showed no toxicity and exhibited a reduced of pulmonary inflammation. When assessed in vitro the smoke from kretek showed less toxic to the cell and less mutagenic (total particulate matter) compared to American-blended cigarettes. In vivo experiments showed kretek cigarette smoke had a lowered toxicity in the respiratory tract^51,53–55^. These cytotoxic studies of kretek seems to be consistent with our current clinical study in the oral cavity. Thus, it could being hypothesised that similar to the evidence found in the toxicity, Indonesian kretek cigarette less cytotoxic to oral mucosal cell lines.

In the current study, we found that alcohol drinking was the factor that most strongly associated to the presence of OPMD. Specifically, respondents who consumed alcohol had an age-standardised 12.2-fold risk of OPMD compared to non-alcohol drinkers. However, multiple logistic regression, which takes into account the combined effects of several independent variables, did not confirm that alcohol drinking was significantly associated with OPMD in our cohort. Many studies have reported alcohol strongly correlated with OC^39,56–58^ with a strong dose–response relationship. These considerations may not be extended to OPMD according to our findings. Arak putih (tuak Dayak) is one of Indonesian traditional homemade alcohol beverage, which contains alcohol typically near 27–34%^59^. The alcohol is fermented from rice. Most of arak putih consumers in this study are from Suku Dayak, Pontianak, WK region. On the other hand, the beer brand consumed in this study is Saguer, local people call the saguer local drinking with ‘beer’ or ‘bir’, this beverage is also traditionally made, and the level of alcohol could possibly reach 70% or more. Because of the high concentration of alcohol in the saguer, some news reported social problem in WP. Most respondents who consumed beer (Saguer) were from WP. Saguer itself originally come from North Sulawesi’s culture and adapted by people from surrounding islands including WP. Saguer is made from fermentation various palm to make sugar^59^.

### Knowledge of OC, oral health behaviour, and OPMD

Inadequate knowledge of OC is a common obstacle for OC awareness and early screening programs^20,60,61^. In the present study, the respondents who had knowledge of OC risk factors had a significantly lower rate of OPMD (OR = 0.2) compared to those who did not, with 82.2% admitting that dentists had never offered any information about OC. The awareness of OC promoted by Government through Indonesian national television and local newspapers was likely to reduce the risk of OPMD, although this trend was not statistically significant in our study.

The lack of knowledge about signs and symptoms of OC leads to a lack of awareness of the need for early screening. Providing written information in the primary care service has proven to be successful to immediately increase knowledge levels of OC, thus resulting in increased likelihood of undergoing OC screening^20,60,61^.

Surprisingly, 6.7% of respondents claimed to never brush their teeth and those who confidently and properly brush their teeth were at a lower risk of OPMD (OR = 0.6). Additionally, the respondents who brush their teeth twice a day have lowered the risk of OPMD compared to those who brush their teeth once a day. This should be taken into consideration when setting proper intervention strategies for raising oral health awareness. A study in Iran reported that there is a significant correlation between the behaviour in maintaining oral health with the behaviour in avoiding oral disease risk factors. Those who monitors their oral health status were more aware of their oral health in general, avoided tobacco use and had better nutrition^62^. Unfortunately, the association between oral health status and non-communicable disease is still largely unappreciated^62,63^.

### Indonesian ethnicity and OPMD

Each year, there are 197 000 deaths, worldwide, from cancer of the mouth and pharynx, with the highest mortality from mouth cancer occurring in Melanesia and south-central Asia^60^. Likewise ethnic differences within countries are at variable risk: for example, a significantly higher number of deaths from OC are recorded in men from the Indian subcontinent in the UK, and OC is more prevalent in areas with a high Asian population^5,64^. Similarly, we have shown that Papua and Dayak^65^ ethnicities have 23.61 and 21.33-fold higher risk of presenting with an OPMD than those of Acehnese ethnicity, Javanese, Balinese, and East Nusa Tenggaranese groups are slightly more likely to have OPMD than Acehnese, whereas Sulawesi and Maluku ethnic groups show a 7-time increased risk compared with Javenese. However, it should be noted that ethnicity was self-reported: Sulawesinese, Malukunese, North Sumatran, West Sumatran, Balinese, and ENT ethnicities were not indigenous to the 5 selected screening regions.

### The association between SES and OPMD

Several factors have been used for SES measurement in relation to OC development^34,66^. In the current study, we measured SES by the location of COE examination, occupation, health insurance, and whether it was easy or not to travel to the dental health facilities. Many studies reported that OC risk is associated with low SES significantly related to lifestyle risk factors^34,67,68^.

The location of COEs were in the community service (CS) and in selected village (VI), in both rural and urban area. CS is the front-line health facility provided by Indonesian government, where most people of the middle and lower SES primarily seek health service as government subsidises the payment^69^. Respondents who were screened at urban areas had a lowered risk (OR = 0.43) of presenting with OPMD compared to those screened at rural areas. This is most likely due to people who living in a rural area may commonly have a lack of hygiene, poor nutrition, bad health facilities, and poor living conditions that may attribute to causation of OC by complex interactions^35^.

Non-government employee had 3.3-fold higher rate of OPMD compared to government employee respondents. The low and unstable income received by non-government employee such as fishermen, traders, and farm workers are the likely reason for poor living conditions and difficulty in buying nutritious food.

Respondents’ wealth can also be assessed by whether they were covered by government insurance or purchased insurance themselves. Indonesian government insurance is strictly only given to those who are of low-income, therefore the respondents who had non-government insurance were considered economically capable for this study. Those respondents who had non-government insurance had a lower risk (OR = 0.33) of OPMD compared to those who had government insurance.

Ability to access dental health facilities is also associated with SES. Citizens with low-income who live in remote areas may experience difficulties in accessing dental health facilities, which can increase their risk of developing oral disease, including cancer^67^. However, this trend was not detected in the current study.

### Study limitations and bias

This observational study is prone to a number of biases: in particular selection bias, measurement bias and recall bias. Low literacy and education levels may limit accuracy of answers. The small sample, especially considering the size of the Indonesian population (273.5 million 2020), limits interpretation.

## Conclusion

The prevalence of OPMD among Indonesians was of surprisingly high (14.3%), with leukoplakia being found to be the most common type of OPMD followed by OSMF, leukoplakia combined OSMF, erytho-leukoplakia, and finally erythroplakia. Smoking, betel quid chewing, and ethnicity were found to be the most significant risk factors for OPMD development, while alcohol drinking was not found to be significant in the multiple logistic regression model. The different ingredients of BQ could well be significantly associated with different rate of OPMD as those who chewed BQ with betel stem inflorescence (OR = 4.2) were found to have a higher risk of OPMD compared to those who chewed BQ with betel leaf. The addition of tobacco to BQ chewing (OR = 10.2) could further increase the risk of OPMD.

In the multiple regression model, other risk factors such as knowledge of OC risk factors, oral health behaviour, and low-income SES group were significantly associated with OPMD occurrence.

Our results suggest an urgent need for primary prevention of OC programs in Indonesia.

We would suggest creating an educational program to help cease habits associated with OPMD/OC, particularly cigarette smoking and BQ chewing.

### Supplementary Information

Supplementary Information.

## Data Availability

The datasets generated during and/or analysed during the current study are available from the corresponding author on reasonable request.

## Abbreviations

μ: Micro
©: Copyright
mg: Milligram
ml: Millilitre
g: Gram
BA: Banda Aceh
BQ: Betel quid
CI: Confident interval
COE: Conventional oral examination
CS: Community service
ENT: East Nusa Tenggaranese
NS: North Sumatra
OC: Oral cancer
OH: Oral Hygiene
OPMD: Oral premalignant disorders
OSMF: Oral submucous fibrosis
PLS: Plain language statement
SD: Standard deviation
SES: Socioeconomic status
VI: Village
WJ: West Java
WK: West Kalimantan
WP: West Papua
