# Supplementary material for: Prevalence and risk factors of oral potentially malignant disorders in Indonesia: a cross-sectional study undertaken in 5 provinces
Source: Sci Rep. 2024 Mar 4;14:5232. doi: 10.1038/s41598-024-54410-4 (PMC10909850; doi:10.1038/s41598-024-54410-4)
Supplement: Supplementary file 1 — Supplementary Information. [file 41598_2024_54410_MOESM1_ESM.pdf]

Prevalence and Risk Factors of Oral Potentially Malignant Disorders in Indonesia: A Large Cross-Sectional Study Undertaken in 5 Provinces

Authors: *Elizabeth Fitriana Sari [1,3], Newell W Johnson[4,5], Michael John McCullough[2], and *Nicola Cirillo[2]

[1]*Dentistry and Oral Health Discipline, Department of Rural clinical Science, La Trobe Rural Health School, Bendigo 3552, Australia*
[2]*Melbourne Dental School, The University of Melbourne, Carlton, VIC 3053, Australia*
[3]*Faculty of Dentistry, Universitas Padjadjaran, 45363, Bandung, Indonesia*
[4]*Menzies Health Institute Queensland, and School of Medicine and Dentistry, Griffith University, Gold Coast, QLD, Australia.*
[5]*Faculty of Dentistry Oral and Craniofacial Sciences, King's College London, London, UK.*

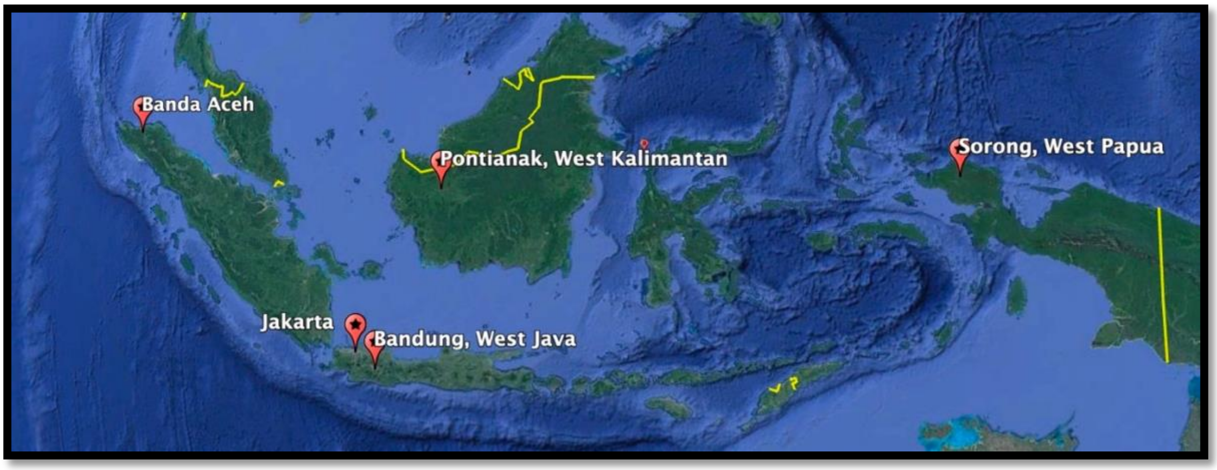

Fig S1. Five selected regions of Indonesia for the study of prevalence and risk factors of oral potentially malignant disorders (OPMD) and oral cancer (OC). These were in Banda Aceh, Bandung (West Java), Jakarta (special city district of Indonesia), Pontianak (West Kalimantan), and Sorong (West Papua). Map data; Google, Image Landsat/ Copernicus).

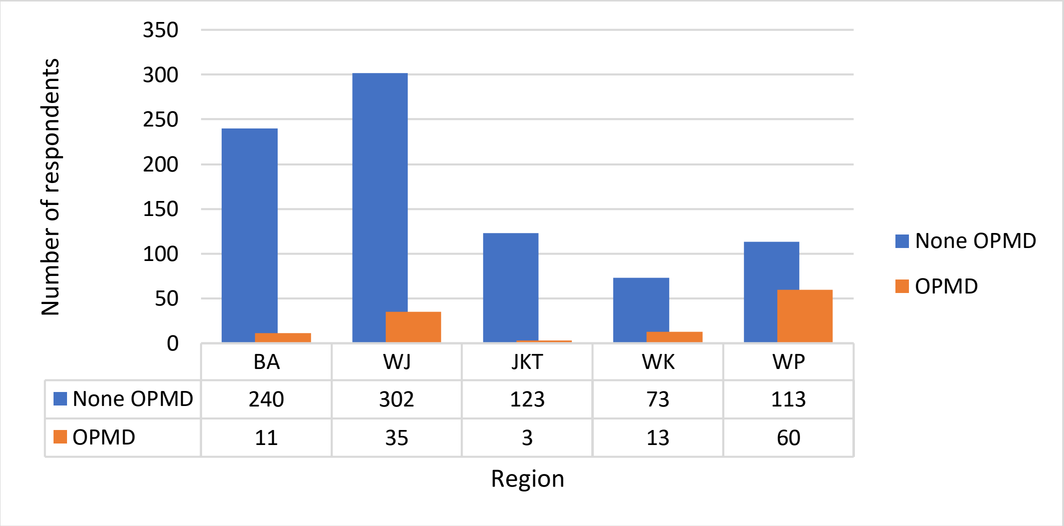

Fig S2. Regional variation in number of OPMD or OC (%)

Supplementary Document 1. Plain language statement
Melbourne Dental School
Faculty of Medicine, Dentistry, and Health Science

Project: **Oral Pre-Malignant and Malignant Lesion Detection Among Indonesian**

Professor Nicola Cirillo
Tel: +61 39341 1473, Email: nicola.cirillo@unimelb.edu.au
Elizabeth Fitriana Sari [DDS, Oral Medicine Specialist]
Email: esari@student.unimelb.edu.au / e.sari@latrobe.edu.au

Introduction
Thank you for your interest in participating in this research project. The following few pages will provide you with further information about the project, so that you can decide if you would like to take part in this research.
Please take the time to read this information carefully. You may ask questions about anything you don't understand or want to know more about.
Your participation is voluntary. If you don't wish to take part, you don't have to. If you begin participating, you can also stop at any time.

What is this research about?
It is predicted that Indonesian have a high number of oral cancer cases as Indonesian people have been exposed many years by oral cancer risk factors such as smoking cigarette, betel nut chewing, and alcohol drinking. However, the number of oral cancer cases among Indonesian people is still unknown. Thus, the purpose of the research project is to get factual number of oral cancer cases among Indonesians, to assess risk factors associated with oral cancer, and to promote awareness by detecting oral cancer early.

What will I be asked to do?
Should you agree to participate after reading this document, you will be asked to indicate your consent by signing infromed consent form. You will than be asked to complete a questionnaire to assess your awareness and risk factors related with oral cancer. To complete the questionnaire will take up to 10 minutes. The next step is, you will undergo a visual and palpation examination on your head, neck and mouth areas to see any abnormalities. For having a detail examination on entire tongue, we will use gauze to retract it gently. All examination steps will not cause any pain and it will only take 5-10 minutes to complete.

What are the possible benefits?
By participating this study, we believe that you will get better understanding about oral cancer and how to prevent it, and as part of this study you will have a free of charge examination for oral cancer detection. Furthermore, If by examination you are suspected to have cancer or pre-cancer condition, than we will reffer you to selected specialist in your region for futher treatments.

There will not any cost arise to take part as participant, either to your insurance or to your own payment. Additionally, our study will not cause any further physical complication, as it is only a simple oral cancer detection.

After completing questionnaire and examination, as our appreciation for your participation, you will be given tools to aid maintaining oral health such as toothbrush and dental paste.

What are the possible risks?
There are no possible risk arise aside of spending extra 15-20 minutes longer than you usually need for your dental treatment, as you need to fill in a questionnaire and undergo examination prior to your dental treatment by your GDP.

Do I have to take part?
No. Participation is completely voluntary. There will not any force to participate this study, and you have the right to both reject and/or join. If you do consent, you also can withdraw your data in the future without any reason. If you wish to withdraw, you can contact responsible researcher or Dr. Elizabeth Fitriana Sari (Email: esari@student.unimelb.edu.au). If you decide to withdraw from the study, our research member will contact you and discuss with you.

Will I hear about the results of this project?
The individual result of this research will be given to you straight away after all research steps have been done completely (questionnaire and examination) together with detail information whether you have suspected lesion or not, and whether you will need further treatment or not.

For the purpose of science, the results of this study will be published on some journals or media and present the results in conferences as poster and/or oral presentation.

What will happen to information about me?
Every collected information and all aspects in this study will be strictly confidential. Only responsible researcher and Dr. Elizabeth Fitriana who have access to the data. We will keep all data securely for a minimum 5 years post publication. Both digital and non-digital data will be kept in the same secure storage or unit of my department. The digital data not intended to be kept will be disposed off by deleting the digital data in SurveyMonkey.com. The paper-based data will also be disposed properly.

Where can I get further information?
If you would like to get more information about the project, please contact the researchers;
A/Professor Nicola Cirillo
Tel: +61 39341 1473, Email: nicola.cirillo@unimelb.edu.au
Elizabeth Fitriana Sari [DDS, Oral Medicine Specialist]
Email: esari@student.unimelb.edu.au

Who can I contact if I have any concerns about the project?
This research project has been approved by the Human Research Ethics Committee of The University of Melbourne. If you have any concerns or complaints about the conduct of this research project, which you do not wish to discuss with the research team, you should contact the Manager, Human Research Ethics, Research Ethics and Integrity, University of Melbourne, VIC 3010. Tel: +61 3 8344 2073 or Email: HumanEthics-complaints@unimelb.edu.au. All complaints will be treated confidentially. In any correspondence, please provide the name of the research team or the name or ethics ID number of the research project.

Supplementary Document 2. Consent form

Melbourne Dental School
Department of Oral Medicine
Faculty of Medicine, Dentistry, and Health Science

Project: **Oral Pre-Malignant and Malignant Lesion Detection Among Indonesian**

A/Professor Nicola Cirillo (Responsible Researcher)
Tel: +61 39341 1473, Email: nicola.cirillo@unimelb.edu.au
Elizabeth Fitriana Sari [DDS, Oral Medicine Specialist] (student)
Email: esari@student.unimelb.edu.au / e.sari@latrobe.edu.au

Name of Participant: ____

1. I consent to participate in this project, the details of which have been explained to me, and I have been provided with a written plain language statement to keep.
2. I understand that the purpose of this research is to investigate oral cancer cases among Indonesian.
3. I understand that my participation in this project is for research purposes only.
4. I acknowledge that the possible effects of participating in this research project have been explained to my satisfaction.
5. In this project I will be required to indicate my consent by signing infromed consent form. I will than be asked to complete a questionnaire to assess my awareness and risk factors related with oral cancer. To complete the questionnaire will take up to 10 minutes. The next step is, I will undergo a visual and palpation examination on my head, neck and mouth areas to see any abnormality. All examination steps will not cause any pain and it will only take 5-10 minutes to complete.
6. I understand that my participation is voluntary and that I am free to withdraw from this project anytime without explanation or prejudice and to withdraw any unprocessed data that I have provided.
7. I understand that the data from this research will be stored at the University of Melbourne and will be destroyed after 5 years post publication.
8. I have been informed that the confidentiality of the information I provide will be safeguarded subject to any legal requirements; my data will be password protected and accessible only by the named researchers.
9. I understand that given the small number of participants involved in the study, it may not be possible to guarantee my anonymity.
10. I understand that after I sign and return this consent form, it will be retained by the researcher.

Participant Signature: __________________________ **Date:** ________________

Supplementary Document 3. Questionnaire for respondents

Personal Data
Name :
Gender :
Date of birth :
Ethnicity :
Monthly income :
Marital status :
Address :
*Personal data will be treated confidentially

Please kindly answer all the questions below.

1. What is the main reason you have come to the GDP today?
    a. Pain
    b. Bleeding
    c. Hole in tooth
    d. Loss teeth
    e. Missing teeth
    f. Sore gums
    g. Other, please specify __________________
2. How long have you had your chief complaint?
    a. 1 day
    b. less than a week
    c. between 1 and 4 weeks
    d. more than a month
    e. more than a year
3. When you have oral problems, where do you go?
   a. GDP
   b. medical doctor
   c. nurse
   d. others, please specify_______________________
4. Is it easy for you to travel to this dental health facility? Yes/No
5. Who will pay for your dental health treatment?
   a. you
   b. insurance (Government/others____________________________________)
   c. other, please specify_____________________
6. Have you ever been diagnosed with oral cancer?
   a. No
   b. Yes, Specify the diagnosis_________________________
7. Has any of your extended family members ever been diagnosed with oral cancer?
   a. No
   b. Yes, Specify the diagnosis_________________________
8. Has any of your friends ever been diagnosed with oral cancer?
   a. No
   b. Yes, Specify the diagnosis_________________________
9. Have you ever heard of someone not known to you being diagnosed with oral cancer from others or from online information?
   a. No

   b. Yes, specify the diagnosis________________________

10. Do you smoke?
    a. No
    b. Yes, please answer the questions below
        - How many cigarettes do you smoke daily? please circle (Less than 10/ between 10 and 20 / more than 20)
        - When did you start to smoke? please circle (in the last 5 years / 5-15 years ago/ more than 15 years ago)
        - What kind of smoke do you inhale? (Pease circle: Filtered / Kretek/ other, please specify_____________)
11. Do you have a habit of chewing betel nuts?
    a. No
    b. Yes, please answer the questions below
        - how many times in a day do you chew? please circle (once a day, 2-5 times a day/ 6-10 times a day / more than 10 times a day)
        - When did you start this habit? (in the last 5 years/ 5-15 years ago / more than 15 years ago)
        - What are the ingredients you put in your betel nuts? Please circle (Betel leaf only / Betel leaf + areca nut + lime / Betel leaf + areca nut + lime + tobacco / others, please specify_____________)
        - Do you prepare it by yourself? Please circle the answer: yes/no
        - Or do you buy it in a package? Please circle the answer: yes/no
12. Do you drink alcohol?
    a. No
    b. Yes, please answer below questions:
        - how many times in a day do you drink? please circle (once a day, 2-5 times a day/ 6-10 times a day / more than 10 times a day)
        - When did you start this habit? (in the last 5 years/ 5-15 years ago / more than 15 years ago)
        - What kind of alcohol do you drink? please specify________________

13. Do you think that there might be other causes of oral cancer apart from smoking, areca nut, and alcohol?
    a. No
    b. Yes, please choose (food/ viruses/ oral sex/ other________________)
14. Do you work mostly exposed by UV?
    a. No
    b. Yes, please answer below question;
        - How many hours roughly in a day?
        - How many days in a month do you work?
15. Do you brush your teeth on a daily basis?
    a. No
    b. Yes
16. How many times do you brush your teeth daily?
17. Do you know how to brush your teeth properly?
    a. No
    b. Yes, please answer the question below:
       Where did you get the information about tooth brushing? (family members/GDPs/teachers at school/ others, please specify___________)

18. How many times do you eat in a day? Please choose: (once a day/ twice a day/ three times a day/ more than three times a day)
19. Do you eat meat every day?
    a. No
    b. Yes
20. Do you eat vegetables every day?
    a. No
    b. Yes
21. Do you eat fruit every day?
    a. No
    b. Yes
22. Have you ever received any information about oral cancer from your GDP?
    a. No
    b. Yes
23. Have you ever received any information about oral cancer from the government?
    a. No
    b. Yes

Table S1. General information

n=973 (100%)	Variable	Frequency	Percentage
Gender	Female	748	76.9
	Male	225	23.1
Occupation	Government Employee	192	19.7
	Non-Government Employee	302	31.0
	Un-Employed	479	49.2
Examination Places	Community Service (rural)	291	29.9
	Community service (urban)	259	26.6
	Villages (rural)	129	13.3
	Village (urban)	294	30.2
Region	Aceh, BA	251	25.8
	Bandung, WJ	337	34.6
	Special district of Jakarta, JKT	126	12.9
	Pontianak, WK	86	8.8
	Sorong, WP	173	17.8
Reason for seeking dentist	Routine consultation	450	46.3
	Dental pain	343	35.3
	Missing teeth	45	4.6
	Periodontal disease	75	7.7
	Other	60	6.2

Healthcare professional sought when having oral problem	Dentist	624	64.1
	Medical doctor	34	3.5
	Nurse	26	2.7
	Treat themselves	164	16.9
	Other	125	12.9
Diagnosed OC previously	No	966	99.3
	Yes	7	0.7
Diagnosed OC in extended family	No	960	98.7
	Yes	13	1.3
Diagnosed OC in Friends	No	959	98.6
	Yes	14	1.4
Diagnosed OC in someone else known from TV, news, internet etc.	No	847	87.1
	Yes	126	13.3

Table S2. Respondents' risk factors of oral cancer and for maintaining oral hygiene.

n=973 (100%)	Variable	Frequency	Percentage
Smoking	No	832	85.5
	Yes	141	14.5
BQ chewing	No	850	87.4
	Yes	123	12.6
Alcohol drinking	No	935	96.1
	Yes	38	3.9
Eat vegetables	No	370	37.8

	Yes	603	62.2
Eat Fruits	No	584	60.0
	Yes	389	40.0
Brush teeth daily	No	62	6.4
	Yes	911	93.6
Brush teeth information	Dentist	364	37.4
	Family	78	8.0
	Teacher at school	79	8.1
	TV	57	5.9
	Other	395	40.6

Table S3. Presence of OPMD, other lesion, distance travelled and insurance of respondents.

**n=973 (100%)**	**Variable**	**Frequency**	**Percentage**
OPMD or OC	No	834	85.7
	Yes	139	14.3
Other oral lesions*	Recurrent aphthous stomatitis	20	
	Frictional keratosis	28	
	Ulcer traumatic	5	
	Benign growth[#]	5	
	Normal	719	
Easy to travel to dental Facilities	No	208	48.2
	Yes	765	78.6

Health Insurance	Government Insurance	403	41.4
	Non-Government Insurance	27	2.8
	Self-Payment	292	30
	Other types	251	25.8
*Three patients had another lesion found besides OPMD, therefore, they have more than one lesion. #These five patients with a benign growth had an exophytic mass covered by normal mucosa that had been present for a considerable time. These lesions were subsequently biopsied.			
